# The Role of Insulation in Patterning Gene Expression

**DOI:** 10.3390/genes10100767

**Published:** 2019-09-28

**Authors:** Isa Özdemir, Maria Cristina Gambetta

**Affiliations:** Center for Integrative Genomics (CIG), University of Lausanne, Genopode Building, 1015 Lausanne, Switzerland; isa.ozdemir@unil.ch

**Keywords:** insulator, gene insulation, IBP, CTCF, gene regulation, genome topology, *Drosophila*, mouse

## Abstract

Development is orchestrated by regulatory elements that turn genes ON or OFF in precise spatial and temporal patterns. Many safety mechanisms prevent inappropriate action of a regulatory element on the wrong gene promoter. In flies and mammals, dedicated DNA elements (insulators) recruit protein factors (insulator binding proteins, or IBPs) to shield promoters from regulatory elements. In mammals, a single IBP called CCCTC-binding factor (CTCF) is known, whereas genetic and biochemical analyses in *Drosophila* have identified a larger repertoire of IBPs. How insulators function at the molecular level is not fully understood, but it is currently thought that they fold chromosomes into conformations that affect regulatory element-promoter communication. Here, we review the discovery of insulators and describe their properties. We discuss recent genetic studies in flies and mice to address the question: Is gene insulation important for animal development? Comparing and contrasting observations in these two species reveal that they have different requirements for insulation, but that insulation is a conserved and critical gene regulation strategy.

## 1. Introduction

During development and cell differentiation, genes are turned ON and OFF in robust spatial and temporal patterns. Gene expression is regulated at multiple levels, a central one being the control of gene transcription. Regulatory elements, such as enhancers and silencers, recruit transcription factors that respectively activate or silence transcription at promoters. In flies and mammals, a developmental enhancer can activate multiple genes [1,2] over large genomic distances, independently of their orientation and position relative to the promoter. This property of enhancers allows promoters to integrate multiple regulatory inputs to achieve refined spatial and temporal expression patterns, but it also leads to potential undesired transcriptional activation of inappropriate genes. This is particularly a concern given the high density of potential regulatory elements found in genomes [3,4]. Several mechanisms limit promiscuous gene regulation. One of them is gene insulation, an activity exerted by dedicated DNA elements (called insulators) and proteins (insulator-binding-proteins, or IBPs) to block the spreading of regulatory element action and thus insulate, or shield, gene promoters from unwanted regulation. The mechanism of insulation is unclear, but it has been proposed to rely on the organization of the chromosomal fiber in three-dimensional (3D) space.

Whereas the importance of enhancers and silencers for controlling developmental gene expression has been demonstrated, the contribution of insulation to this process is less clear. This review discusses the relevance of insulation for animal development. We first review the discovery of insulators and their binding proteins (Section 2) and describe their unique properties (Section 3). We then compare and contrast recent studies that have genetically engineered mice and flies to address the developmental roles of insulators and IBPs in these two divergent models (Section 4). We extract key concepts and highlight open questions regarding how insulators regulate genes (Section 5).

## 2. Discovery of Insulators and their Binding Proteins

### 2.1. Discovery of Insulators

An early hint that insulators exist in the *Drosophila melanogaster* genome to maintain the independence of neighboring regulatory domains came from studies of the *Hox* gene, *Abdominal-B* (*Abd-B*). A spontaneous chromosomal deletion removed the boundary between two enhancer domains that each drive *Abd-B* expression in separate body segments. This deletion resulted in *Abd-B* activation by the wrong enhancer in the wrong body segment, as if the two adjacent enhancer domains had lost their abilities to act independently in different segments (discussed further in Section 4.2.1.) [5]. Additional evidence for insulators came from studies of the mutagenic effects of the *gypsy* transposable element in flies. When *gypsy* transposed in between a gene and its distal tissue-specific enhancers, it prevented gene expression only in this respective tissue [6,7,8], suggesting that *gypsy* somehow insulated the promoter from its distal enhancer.

**Figure 1:**
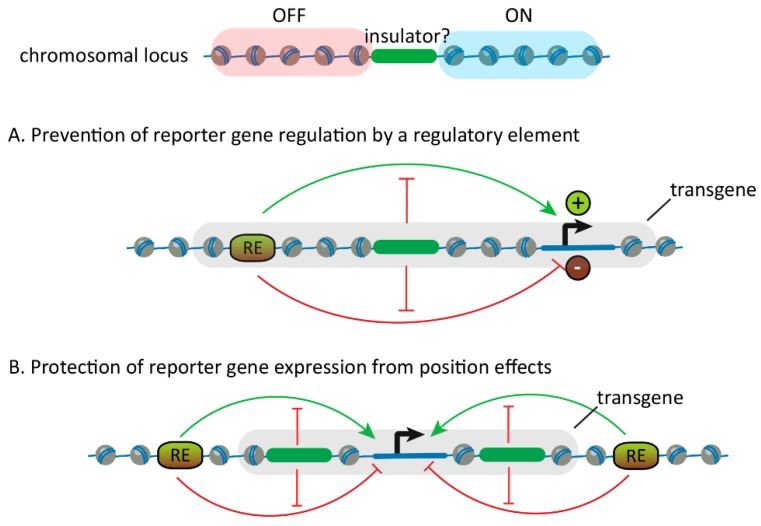
Insulator reporter assays. (Top) Candidate insulators (green) have been selected based on their locations between chromosomal loci (depicted as a string of nucleosomes) with different transcriptional states and tested in insulator reporter assays. (**A**) In one assay, the candidate insulator is placed between a regulatory element (RE) and a reporter gene in a transgene (grey). If it is an insulator, the reporter gene will not be regulated (activated or silenced) by the regulatory element. (**B**) In another assay, a transgene (grey) containing a reporter gene (black arrow) is flanked by candidate insulators (green) and inserted in random positions in the genome. If the candidate sequences are insulators, the reporter gene will be expressed at similar levels in different locations because it will be shielded from the influences of local regulatory elements (referred to as position effects).

Using a newly developed reporter assay, the first genomic regions were tested for insulator activity in flies in 1991. Test loci were selected based on their locations between transcriptionally active and inactive genomic regions. The first loci tested were the specialized chromatin structure (*scs* and *scs’*) insulators that flank the heat-shock gene locus that becomes “puffed” upon heat-shock induction [9,10]. Likewise, in vertebrates, the first candidate insulator was the chicken *5’HS4* insulator located near the boundary between the decondensed beta-globin locus expressed in erythroid cells and a chromatin domain that remains condensed in those cells [11]. These assays confirmed that the tested loci could protect a reporter gene promoter from activation/silencing by an enhancer/silencer when interposed [10,11] (Figure 1A). Also, candidate insulators flanking a transgene ensured that the transgene inserted at random chromosomal locations was expressed at similar levels by shielding them from local regulatory elements [9] (Figure 1B). 

It is important to note that while many studies have characterized insulator function in reporter gene assays, only a small number of selected insulators have been functionally assessed in their endogenous loci. Also, insulators that have been characterized to date have all been hand-picked for study [12,13]. Therefore, important questions remain about how frequent and diverse insulators are in genomes.

### 2.2. Discovery of Insulator-Binding Proteins (IBPs)

**Figure 2:**
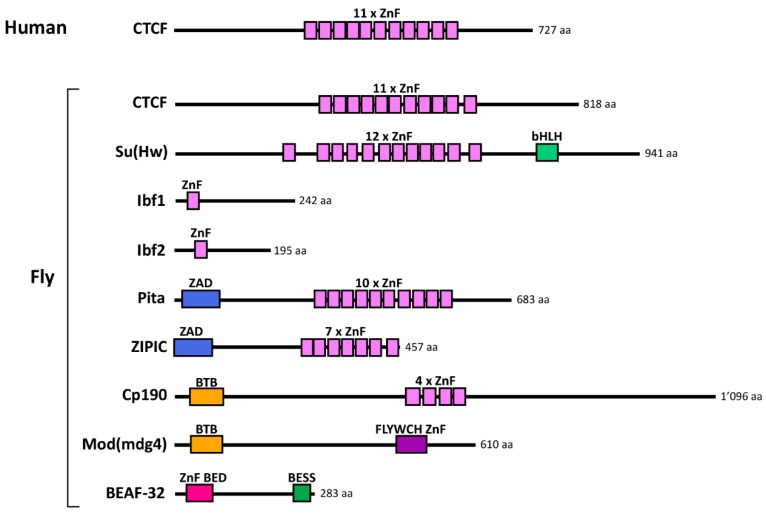
Examples of known insulator-binding proteins (IBPs) in humans and flies. Schematics of the protein domain organization of IBPs are drawn to scale. Abbreviations are as follows: CTCF: CCCTC-binding factor; Su(Hw): Suppressor of Hairy wing; Ibf: Insulator binding factor; ZIPIC: Zinc-finger protein interacting with Cp190; Cp190: Centrosomal protein 190 kDa; Mod(mdg4): modifier of mdg4; BEAF-32: Boundary element-associated factor of 32 kDa; ZnF: zinc finger; ZAD: zinc finger associated domain; BTB: Broad-Complex, Tramtrack and Bric-a-brac; bHLH: basic helix-loop-helix; BED: BEAF-32 and DREF; BESS: BEAF-32, Suvar(3)7 and Stonewall.

In flies and mammals, many characterized insulators are a few hundred base pairs long and are thought to act by recruiting insulator-binding proteins (IBPs). The first IBP to be discovered was CCCTC-binding factor (CTCF). CTCF was purified from chicken cell extracts through its interaction with the beta-globin insulator [14] (Figure 2). To date, CTCF remains the major protein implicated in insulation in vertebrates. In addition, certain sites in the mammalian genome bound by RNA polymerase III have been shown to be insulators [15], suggesting that other insulators also exist outside of IBP binding sites.

In contrast to vertebrates, experiments in *Drosophila* have identified a dozen or more proteins with insulator activity (see selected IBPs in Figure 2). *Drosophila* CTCF was identified as the homolog of vertebrate CTCF, and its insulator function was conserved in a reporter assay [16]. CTCF and its recognition site in DNA are in fact present in many bilaterian animals but absent from fungi and plants [17,18]. Other fly IBPs were identified (1) based on their ability to bind to characterized insulators and mediate their function [8,19,20], (2) in genetic screens as being required for the function of a specific insulator [21,22], and (3) more recently in biochemical purifications of a specific IBP called Centrosomal protein 190 kDa (Cp190) [23,24,25]. It is important to note that the insulator activity of several IBPs has only been characterized in transgenic reporter assays, so evidence that these proteins insulate endogenous genes is mostly still lacking. Several IBPs bind directly to DNA through zinc finger (ZnF) domains (Figure 2). Fly IBPs except CTCF do not seem well conserved in evolution [18,26,27]. Flies may have more IBPs than vertebrates because of a possibly greater need for gene insulation in a compact genome. It is, however, also possible that the greater number of functional assays performed in flies simply allowed deeper sampling of IBPs. Fly IBPs are modular proteins and mammalian proteins with conserved protein domain organizations do exist. It remains to be determined whether functional homologs of fly IBPs exist in other species.

## 3. Properties of Insulators and their Binding Proteins

IBPs bind to many sites in the genome. For example, fly and mammalian CTCF binds to thousands of sites that are often intergenic and invariantly bound in different cell types [28,29]. A minute fraction of these sites has been tested for insulator activity. Here, we describe the intriguing properties ascribed to mammalian and fly insulators.

### 3.1. Blocking the Communication Between Regulatory Elements and Promoters

**Figure 3:**
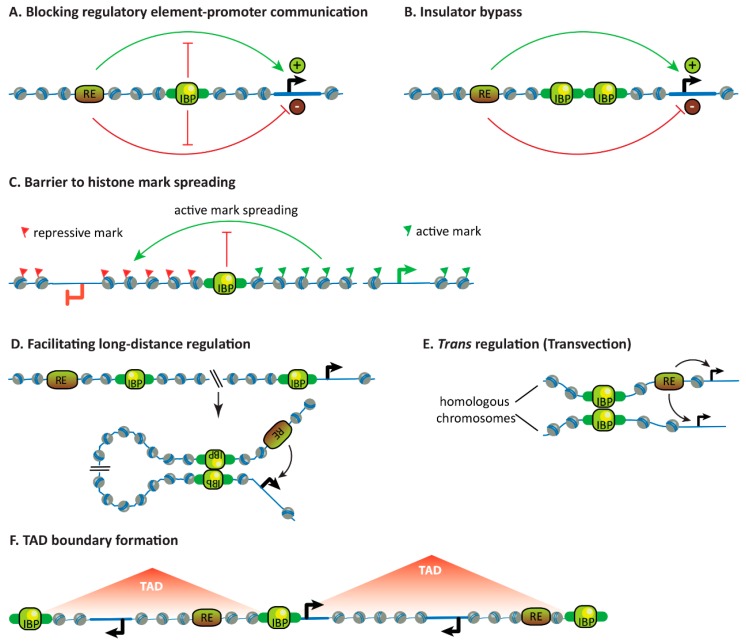
Properties of insulators and their binding proteins. Insulators may block the communication between regulatory elements and promoters (**A**), support bypass when paired (**B**), act as barriers to histone mark spreading (**C**), facilitate long-distance communication between a regulatory element and a promoter in *cis* (**D**) and in *trans* (**E**), and form boundaries of Topologically Associated Domains (TADs) (**F**). Some of these properties have so far only been described in flies (B and E) and others only in mammals (**F**).

As previously mentioned, insulators are defined by their ability to block the communication between enhancers or silencers and gene promoters when interposed [9,10,30,31] (Figure 3A), despite the fact that enhancers and silencers regulate transcription through very different mechanisms. Insulators do not affect the individual functionalities of these elements (i.e., their abilities to regulate or be regulated by, respectively, other elements on opposite sides of the insulator [32]) and they have no enhancer or silencer activity of their own. More generally, insulators are thought to delimit domains of independent gene regulation. 

In the few studies to date in which insulators have been analyzed in situ, deletion of endogenous insulators in mammals and flies often resulted in ectopic activation of a gene by a formerly insulated enhancer. This is referred to as enhancer-adoption or enhancer-hijacking [33]. There are much fewer examples of mammalian insulators shielding genes from ectopic silencers, though this has been observed in flies (see Section 4). Clearly, however, not all IBP binding sites are insulators because many examples are known in which regulatory elements regulate genes beyond IBP-bound sites in flies and mammals. The context-dependent functions of insulators are not well understood, but partly depend on genomic location, as shown for example in flies, in which an insulator reporter transgene shows different insulator strengths when transposed into different loci [12].

### 3.2. Insulator Bypass

Bypass is the property of fly insulators by which two tandem copies of insulators cancel their activities and no longer block the communication between regulatory elements and promoters when interposed [34,35] (Figure 3B). In some cases, bypass is only effective between pairs of insulators in specific orientations [36]. Analogous insulator bypass experiments have not been performed in mammals.

### 3.3. Forming Barriers to Histone Mark Spreading

Some insulators lie at borders between different chromatin landscapes and may block the spreading of histone modifications (Figure 3C). This has been observed for insulators of *Hox* genes in mouse cells [37] and fly embryos [38,39,40]. Yet, insulators do not globally function as barriers to histone mark spreading because they are not enriched at these borders. In mammalian cells, only ~10% of lamina-associated domain (LAD) borders are bound by CTCF [41] and less than 5% of Polycomb-repressed domains marked by trimethylated lysine 27 on histone H3 (H3K27me3) are bordered by CTCF [42]. In *Drosophila* cells, knock-downs of IBPs in cultured cells have led to conflicting results regarding changes in histone mark distributions, but IBPs are also unlikely to globally regulate, for example, H3K27me3 domain boundaries [12,43].

### 3.4. Facilitating Long-Distance Gene Regulation

Though many enhancers act locally, developmental enhancers can act over large distances in mammals (up to over 1 Mb) and flies (up to 80 kb at the *cut* locus) [7,44,45]. Enhancer activity decays over increasing distances [1,46], so how do enhancers find their long-distance targets? The dominant view is that enhancers influence transcription from promoters through proximity in 3D space (though other mechanisms of enhancer action may also be relevant [45,47]). This is supported by chromosome conformation capture, microscopy and recent orthogonal techniques revealing that co-regulated enhancers and promoters often come together in hubs [48,49,50,51].

Though it may seem contradictory given their characteristic ability to block the communication between regulatory elements and promoters, insulators may also foster long-range regulation. In mice, CTCF may bridge certain developmental enhancers and promoters to enable their communication (see Section 4.1.2.). 

It is less clear whether insulators support long-range gene regulation in flies. On the one hand, there are clear examples of genomic loci that can both act as insulators and support long-distance regulation in *Hox* genes (see Section 4.2.1.) and at the even-skipped (*eve*) locus [52]. At *eve*, two insulators have been characterized that are capable of highly specific pairing with themselves or with each other when they are placed in specific orientations on the same chromosome or on homologous chromosomes [53]. Positioning these insulators in specific orientations can block some regulatory interactions while facilitating others. The physical pairing of *eve* insulators placed more than 140 kb apart was visualized in live fly embryos and preceded transcriptional activation of a reporter gene linked to one insulator by distal enhancers linked to the other insulator, indicating that insulator pairing mediated long-distance enhancer-promoter communication [54]. Importantly, however, the ability of *Hox* gene boundaries to support long-distance regulation has been shown to be separable from their insulator activity and independent of IBP binding (see Section 4.2.1.). On the other hand, other studies have observed that distantly placed and specifically oriented IBP binding sites in reporter transgenes can pair and bridge an upstream enhancer or silencer to a downstream promoter and enable distal regulation [55,56]. This was, in some cases, shown to occur through formation of an observable chromosomal loop [57]. In brief, whether long-distance pairing and insulating activities of insulators are intrinsic or separable activities remains to be clarified – for example, by asking whether long-distance gene regulation is compromised in IBP mutant *Drosophila*.

### 3.5. Facilitating Trans-Regulation

Transvection is a phenomenon described in flies in which regulatory elements on one chromosome regulate a gene on the homologous chromosome [58]. Transvection has been observed at many endogenous developmental loci and depends on homologous chromosome pairing [59], which becomes pervasive in *Drosophila* after early embryogenesis [60]. Insulators further facilitate transvection by possibly stabilizing homolog pairing [30,61]. When transvection was imaged live in fly embryos, insulators present on both homologous chromosomes allowed an enhancer to almost equally activate a gene in *cis* and in *trans* and increased the stability, though not the frequency, of pairing of these loci in very early embryos when homolog pairing is still inefficient [62].

In mammals, *trans*-homolog regulation has not been so clearly demonstrated, likely because homologous chromosome pairing is not as pervasive as in flies.

### 3.6. Influencing Chromosome Topology

Chromosomes are partitioned into contiguous and often cell-type invariant Topologically Associated Domains (TADs) in many organisms including flies and mammals. TADs are visualized in Chromosome Conformation Capture (3C) techniques like Hi-C that measure chemical crosslinking frequencies between distant genomic loci as a proxy for their proximity in 3D space [63,64,65].

In mammals, CTCF plays a central role in forming the boundaries of TADs and other chromosomal loops. Chromosomal loops of various sizes are widespread in mammalian Hi-C maps, and TADs are in fact the larger of these loops. Loops are believed to be formed by extrusion of chromatin by cohesin until cohesin reaches a pair of convergently oriented CTCF-bound sites [66,67,68]. This model is supported by compelling experimental evidence: (1) Convergently oriented CTCF-bound sites are present at the majority of loop anchors [69,70], (2) deletion of a CTCF site can result in fusion of two adjacent loops [37,66,71,72], (3) loops can be rewired or created by precise inversions or insertions of CTCF sites [73,74], and (4) modulation of CTCF or cohesin levels on chromatin dramatically affect loops genome-wide [68,75,76,77,78]. Within TADs, nested chromosomal loops established by CTCF can link enhancers and promoters constitutively or in an activity-dependent manner [69,79]. The gene regulatory functions of mammalian TADs are discussed in Section 4, but they are generally viewed as basic structural and functional units in which genes are coordinately regulated [64,80]. It is important to note that CTCF and cohesin are not the only architectural proteins, and transcription-related processes also drive genome folding and compartmentalization in mammals [81].

The folding principles of the *Drosophila* genome appear different from mammals. It is not clear whether TADs arise from loop extrusion and whether CTCF or other IBPs participate in this process [82]. Earlier locus-specific studies had suggested that insulators loop towards each other [83,84], yet recent Hi-C studies in flies have not observed widespread insulator-anchored chromosomal loops. Fly IBPs and/or their motifs in DNA seem enriched at a subset of TAD boundaries but the extent of this enrichment varies greatly from study to study (for example, from about a quarter of TAD boundaries in one analysis [85] to >90% in another [86]). Most *Drosophila* TADs do not have focal peaks at their corners like many mammalian TADs do [87], chromosomal loops are rare (a few hundred loops are visible in *Drosophila* Hi-C maps [87,88,89] compared to ~10,000 loops detected in human cells [69]), and neither CTCF nor IBPs are enriched at loop anchors [81,87,89]. Therefore, whether and how IBPs form TAD boundaries or other structures is currently unclear, and their contribution has been questioned [81,90,91]. Rather, transcription-related processes such as histone modifications or the recruitment of the transcriptional machinery to chromatin have been proposed to be major drivers of 3D genome organization in flies. Indeed, TAD boundaries are enriched at transitions between chromatin domains with different histone modifications or at divergently transcribed gene promoters [81,83,91,92]. Moreover, Hi-C experiments performed in early fly embryos before the onset of transcription [93,94] or in fly cells in which transcription was globally perturbed [81,82,95] have reported global effects on genome architecture. Nevertheless, topological boundaries exist even within chromatin domains with the same transcriptional and epigenetic state, therefore, transcription-independent mechanisms that form TAD boundaries must exist [96]. Future experiments will reveal what these mechanisms are and whether IBP mutants display topological defects. 

## 4. Relevance of gene insulation for animal development

### 4.1. In Mammals

#### 4.1.1. CCCTC-Binding Factor (CTCF) is Essential in Mammalian Cells

CTCF is essential for the viability of mammalian cells, including mouse embryonic stem cells (mESCs) [75,97] and many other cell types [98,99]. CTCF knock-out mice undergo apoptosis at the peri-implantation stage, one day after maternal CTCF becomes undetectable [100]. In other cell types like immune cells, CTCF is required for cell cycle progression and differentiation [101,102]. The inability to culture cells lacking CTCF has limited studies to observing early effects after acute CTCF depletion or to locus-specific perturbations of CTCF binding sites or entire TAD boundaries. Despite these technical challenges, much of what we currently know about CTCF function comes from mechanistic studies in mammalian cells. 

CTCF binds pervasively in the genome [80,103] and was assumed to globally affect transcription. Acute depletion of CTCF in mESCs resulted in a dramatic loss of CTCF/cohesin-anchored chromosomal loops, including TAD boundaries, but only limited effects on global messenger RNA (mRNA) levels. Specifically, only ~400 genes were differentially expressed one day after depletion, and ~4,000 were affected four days after depletion which might include indirect effects of CTCF depletion on gene transcription [75]. Upregulated genes tended to be closer (within 200 kb) to active enhancers from which they were normally separated by a TAD boundary [75], which could be evidence for CTCF’s enhancer-blocking function. Yet, the discordance between the magnitude of effects of CTCF depletion on genome topology and gene regulation indicated that current thoughts about how chromosomal folding impacts gene expression are incomplete.

The manipulation of specific CTCF binding sites or broader regions have revealed that CTCF insulates critical developmental genes including the imprinted *Igf2/H19* genes [104], *Hox* genes [37,105], pluripotency genes [106,107] and oncogenes [71]. Below, we discuss recent studies in which specific CTCF sites or TAD borders were disrupted in mice and how this affected developmental gene regulation.

#### 4.1.2. CTCF-Mediated Chromosomal Loops Foster Long-Range Gene Regulation

Compared to flies, mammals have a ~15-fold larger genome for only ~1.5-times more genes. Many mammalian developmental genes lie in large gene deserts comprising enhancers hundreds of kb away. Analyses of hundreds of enhancer trap insertions in mouse embryos revealed that enhancers are active in broad domains, typically a few hundred kb long, that correlate strikingly well with TADs [108]. Another configuration is observed at the mouse *HoxD* gene cluster which lies at the boundary between two TADs that each drive expression of specific *HoxD* genes in the proximal part of limbs or in digits, respectively [109,110]. Interestingly, many CTCF sites present in these TADs seem placed and oriented in functionally relevant ways. At *HoxD*, clustered CTCF-bound sites point from the gene cluster towards convergently oriented CTCF sites located near enhancers within the flanking TADs, as well as at the flanking TADs’ boundaries [111]. Do TADs bridge target genes to their enhancers to enable regulation?

One example supporting this notion is the Sonic hedgehog (*Shh*) gene locus encoding a morphogen important for limb and brain development. *Shh* and its enhancers are comprised within a TAD [112], including the ZRS enhancer (zone of polarizing activity regulatory sequence) located 850 kb away that drives *Shh* expression in the developing limb bud [113]. Chromosomal rearrangements that changed the linear distance between *Shh* and the ZRS within the TAD did not affect *Shh* expression, but others that placed *Shh* and the ZRS in separate TADs disrupted *Shh* expression in limbs, resulting in malformations [112]. This indicated that within TADs, genomic distances have minimal effects on enhancer-promoter interactions, whereas comparable or even smaller linear distances became prohibitive outside of TADs. A possible mechanistic explanation is that loop extrusion by cohesin between CTCF-bound TAD boundaries may allow an enhancer to sample promoters within a TAD.

Within TADs, CTCF-anchored chromosomal loops can bridge a gene to its enhancers. Some of these loops are constitutive (present in cells in which the gene is ON and in cells in which it is OFF) and others are tissue-specific [109,110,114]. An example of a constitutive loop occurs between the *Shh* and the ZRS, which are in enhanced proximity (even closer than other loci within the same TAD) in many mouse tissues [115]. This constitutive topology does not result in ubiquitous gene activation, but does it facilitate ZRS regulation of *Shh*? Precise deletions of individual CTCF binding sites near *Shh* and/or the ZRS caused *Shh* and ZRS to move further apart, but *Shh* transcription was still activated in limbs [116] albeit in some cases at only 50% of wildtype levels [117]. These mutations did not cause a phenotype except in a sensitized genetic background [117]. This contrasts with the stronger phenotypic effects of chromosomal rearrangements placing *Shh* and ZRS on opposite sides of a TAD boundary (discussed previously). Pre-formed CTCF-anchored topologies are therefore not strictly required for enhancer-promoter communication, but they may make this communication more robust. A similar observation was made in the *Sox9* TAD. *Sox9* enhancers could still activate *Sox9* transcription in limb buds of mice in which all CTCF sites, both inside the *Sox9* TAD and at its border with the neighboring *Kcnj2* TAD were deleted resulting in TAD fusion. *Sox9* levels were only reduced by ~10% without any phenotypic consequence [118].

In another example, a CTCF-mediated topology supported gene activation by a long-distance enhancer in an ectopic context. Chromosomal inversions engineered in mice near the *Epha4* gene locus containing a potent enhancer cluster and adjacent CTCF binding sites resulted in formation of “architectural stripes” visualized by Hi-C that emanated from the clustered CTCF sites at the base of the enhancer cluster [119]. Ectopically-activated genes in each inversion contacted the architectural stripe [119]. The extent of the stripe was similar in inversions with different breakpoints, suggesting a comparable distance-decay trend. Taken together, these studies reveal that CTCF-mediated topologies can delimit the search space between enhancers and promoters to reinforce regulatory interactions.

#### 4.1.3. The Role of Insulation at Topologically Associated Domain (TAD) Boundaries

**Figure 4:**
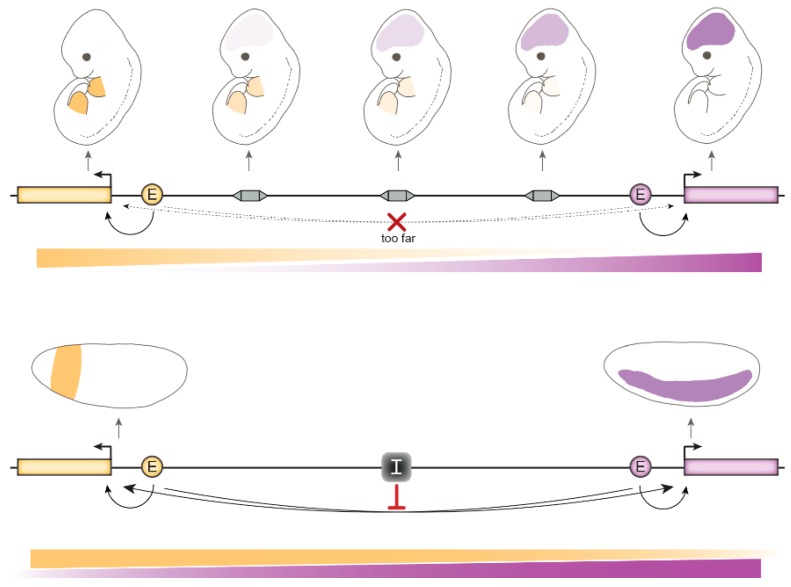
An important difference between mammals and flies is the greater distances between developmental genes and their regulatory elements in mammals, whereas the fly genome is more compact. This may result in a greater need for insulation in flies. (**Top**) Two genes (orange and purple) are activated by their respective enhancers in non-overlapping mouse tissues (limb buds and brain in this example). Enhancer traps (grey rectangles) reveal weakening of enhancer activities with increasing distances from these enhancers. The two enhancers are too far apart to cross-regulate their non-cognate genes. Enhancer strength at different distances is represented underneath. (**Bottom**) Two genes are activated by their respective enhancers in non-overlapping cells in fly embryos, but these are close enough to potentially result in cross-regulation of one gene by the other’s enhancer. Therefore, an insulator (grey box labelled “I”) is necessary to maintain independent gene expression patterns.

Large genomic distances between adjacent developmental genes are in many cases sufficient to prevent interactions between genes and non-cognate enhancers without the need of insulators (Figure 4). This is the case of the neighboring *Nkx2-2* and *Pax1* developmental regulator genes that are expressed in different patterns in mouse embryos. A survey of the regulatory landscape between these genes using enhancer trap insertions did not provide evidence for the presence of an insulator between them. Instead, the natural decay of each gene’s enhancer activities with increasing distance was sufficient to prevent cross-regulation [120]. How important, therefore, is insulation in the mammalian genome?

TADs boundaries appear to generally delimit enhancer function since enhancer activity is detected at many locations within a TAD but not beyond its boundaries [108,112]. Large chromosomal rearrangements that disrupt TADs have been observed to lead to ectopic activation of developmental genes by formerly insulated enhancers, resulting in developmental defects in mice and humans [33,121] or cancer [71,122]. In these cases, TAD boundaries seem crucial for isolating regulatory domains.

Yet, more precise manipulations of individual CTCF-binding sites have led to a spectrum of effects, from strong to undetectable. This is, for example, illustrated at *Hox* gene clusters (*HoxA*, *HoxB*, *HoxC* and *HoxD*) that harbor CTCF binding sites. On the one hand, deletions of specific CTCF binding sites between independently regulated *Hox* genes in *HoxA* or *HoxC* led to homeotic transformations of mice skeletons [105]. On the other hand, in a different developmental context – that of mouse limb development – deletions of CTCF sites at *HoxD* had mild effects [111]. CTCF binding sites within *HoxD* form a boundary between the proximal limb and digit regulatory TADs mentioned before. Deletions of even large portions of *HoxD* encompassing several CTCF sites in many cases did not result in ectopic *HoxD* gene activation by enhancers from the wrong TAD, even though increasingly larger deletions did end up leading to noticeable ectopic activation [111].

**Figure 5:**
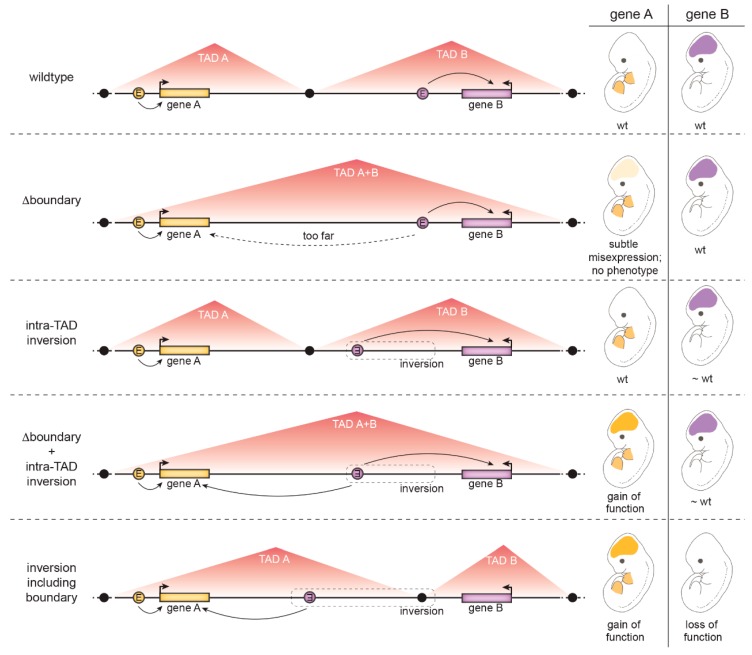
Re-wiring of enhancer-promoter interactions requires loss of insulation and enhancer re-direction, as illustrated at a hypothetical model locus. (Row 1) A model locus spans two TADs, each containing a developmental gene (orange or purple) and their respective enhancers that drive each gene’s respective wildtype (wt) expression pattern (in embryonic limb buds or brains in this example). (Row 2) Deletion of a TAD boundary only leads to weak activation of a formerly insulated gene by the other gene’s enhancer (in this case resulting in weak expression of gene A in the brain) because this enhancer is far away, resulting in no phenotypic outcome. Note that in addition to deleting CTCF binding sites at the TAD boundary, additional CTCF binding sites within TAD B (not shown) must be deleted to result in TAD fusion. (Row 3) An intra-TAD inversion does not affect gene activation because enhancer-promoter distances are relatively insensitive to changes in linear distances within the original TAD. (Row 4) Deletion of a TAD boundary combined with an inversion that moves gene B’s enhancer closer to gene A results in strong ectopic activation of gene A in gene B’s pattern and a gain-of-function (GOF) phenotype. (Row 5) An inversion involving the TAD boundary can place enhancer B in proximity to gene A, resulting in ectopic expression of gene A in gene B’s pattern and gene A GOF. At the same time, gene B is now insulated from its enhancer and is no longer activated in its endogenous pattern, leading to gene B loss-of-function in this tissue.

It may seem paradoxical that in several reported cases, the severe pathological effects of chromosomal rearrangements breaking TADs were not recapitulated by smaller rearrangements [123] or manipulation of individual CTCF binding sites [116]. For example, the deletion of several CTCF sites that separate the *Kcnj2* and *Sox9* genes, which are expressed in different cells in developing mouse limbs, caused weak ectopic activation of *Kcnj2* transcription in a *Sox9* pattern but no phenotype [118]. Why do precise manipulations fail to reproduce phenotypes observed in larger chromosomal rearrangements? One possibility is that not only loss of insulation, but additionally rearranged proximities between non-cognate enhancer-promoter pairs, are necessary to achieve significant ectopic gene activation. A series of configurations of the *Kcnj2/Sox9* locus were genetically engineered in mice to separately test the relevance of insulation at CTCF binding sites on one hand, and of rearranged distances between putative enhancers and the *Kcnj2* and *Sox9* genes on the other. Only the combination of both CTCF site deletions and intra-TAD rearrangements led to severe gene misexpression and developmental phenotypes [118] (Figure 5).

Recent studies are uncovering several reasons that together may explain why the insulator activities of individual CTCF binding sites may be masked in genome-engineering experiments in mammalian cells. This highlights the multiple levels at which the communication between regulatory elements and gene promoters is controlled in mammals. These levels of control can be divided into two main categories: mechanisms that confer robustness to genome topology (points 1–4 below) and mechanisms that limit promiscuous enhancer-promoter communication between regulatory elements and genes that are in proximity (points 5–8 below).

1. TAD boundaries are in several cases composed of many clustered CTCF binding sites in convergent orientations facing opposite TAD borders. Several CTCF sites therefore have to be deleted to weaken the boundary. A clear example discussed above is the *HoxD* boundary in mice: only a ~400 kb deletion including the whole *HoxD* cluster resulted in TAD boundary loss and fusion of the flanking TADs [111].
2. TADs are hierarchical structures composed of nested smaller CTCF-mediated loops [124]. Therefore, enhancers and promoters are not only connected by looping between TAD borders but additionally by intra-TAD loops. For example, at the *Kcnj2/Sox9* locus, deletion of CTCF sites at the *Kcnj2/Sox9* TAD boundary was not sufficient for the TADs to fuse – all major CTCF sites in the Sox9 TAD had to be additionally removed [118].
3. Deletions of CTCF sites can lead to rearranged contacts with other pre-existing and even previously unoccupied or weakly bound CTCF sites [117,118].
4. Additional CTCF-independent forces drive genome compartmentalization, such as the segregation of transcriptionally active and silent chromatin or the yet not-well understood property of chromatin domains with specific histone marks to coalesce [69,81]. Thus, topology is not completely abrogated by CTCF manipulation [75].
5. Different compatibilities exist between promoter and enhancer types, which explains why some genes are activated by only specific enhancers [125,126].
6. Activation of a gene by an ectopic enhancer may depend on its chromatin properties. For example, H3K27me3-decorated genes responded more strongly to an ectopic enhancer placed in proximity by a chromosomal inversion [119].
7. The presence of a “decoy promoter” that competes for enhancer activity may mask ectopic activation of a gene [127].
8. Even measurable changes in gene transcript levels are not always sufficient to cause an observable effect on gene function [117].

In brief, TADs and other CTCF-mediated topologies provide robustness to gene expression. In specific, but not all genomic contexts, insulation is critical for developmental gene regulation.

### 4.2. In Flies

#### 4.2.1. Developmental Roles of Fly Insulators

**Figure 6:**
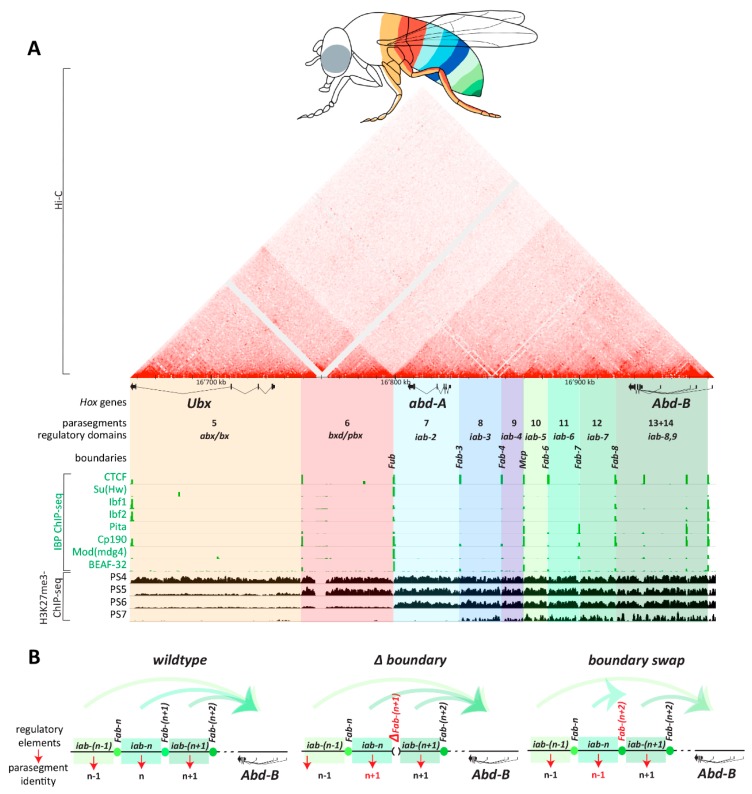
The importance of fly insulators in *Hox* gene expression. (**A**) Map (dm6 coordinates indicated) of the bithorax complex (BX-C) in *Drosophila melanogaster* containing the *Ultrabithorax* (*Ubx*), *abdominal-A* (*abd-A*) and *Abdominal-B* (*Abd-B*) genes (rectangles indicate exons, connecting lines indicate splice junctions, and arrowheads point in the direction of transcription). Genetically-identified regulatory domains (colored) driving expression of a *Hox* gene in a specific body parasegment and the boundaries between these domains are indicated. Published chromatin immunoprecipitation (ChIP)-seq tracks [23,24,149,150,151] of several IBPs shows colocalization with boundaries. Parasegment-specific H3K27me3 profiles show domain-wide loss of H3K27me3 in body segments in which a respective regulatory domain is active [38]. A Hi-C map generated in Kc cells [89] (Top) show that some regulatory domains correspond to TADs, with a very clear separation of *Ubx* regulatory regions from *abd-A* and *Abd-B* regulatory regions. (**B**) Summary of genetic boundary deletion and swapping experiments, using generic *Abd-B* boundaries as examples (*Fab-n* depicts the boundary between *iab-(n - 1)* and *iab-n* regulatory domains that pattern body segments n-1 and n, respectively). (Middle) When a boundary is deleted, enhancing activities of the two formerly insulated regulatory domains mix, and segment n transforms its identity to that of segment *n* + 1 (regulatory domains are thought to act in an additive fashion [152]). (Right) When a boundary is swapped for a heterologous insulator (in this example, for the next boundary *Fab-(n + 2)*), the flanking regulatory domains remain insulated, but the preceding regulatory domain is typically no longer able to bypass the heterologous boundary. This leads to the transformation of the identity of segment *n* to that of *n* − 1.

One of the best understood developmental roles of fly insulators is to regulate *Hox* gene expression along the anterior-posterior body axis. For *Hox* genes of the bithorax complex (BX-C) that specify the identities of thoracic and abdominal segments, independent regulatory domains containing enhancers and silencers drive expression of their respective *Hox* gene in specific body segments [128] (Figure 6A). Genetically-identified boundaries flank each regulatory domain and insulate them functionally and physically. CTCF binds together with other IBPs at these boundaries (Figure 6A). When a boundary is deleted, the two flanking regulatory domains fuse into a new unit in which the activities of both their enhancers and silencers mix [129] (Figure 6B). BX-C *Hox* boundaries also coincide with topological boundaries between TADs that span regulatory domains, and boundary deletion results in TAD fusion [96]. The insulator activity of Hox boundaries depends on IBPs [128,129], but it is not yet known whether topological defects arise in IBP mutants.

The regulatory domains located distally from *Hox* genes regulate them across large distances up to 50 kb, and paradoxically they must overcome intervening boundaries to regulate their respective *Hox* promoter. Genetic experiments swapping *Hox* boundaries for each other and with unrelated insulators have revealed that the insulator (enhancer-blocking) function of different boundaries is generic, but swapped boundaries frequently fail to support long-range regulation of *Hox* genes by their respective regulatory domains [130,131,132,133,134] (Figure 6B). In one case, a boundary from the *Abd-B* gene swapped into the boundary separating *abd-A* and *Abd-B* regulatory domains ectopically directed an *abd-A* regulatory domain (*iab-4*) to regulate the *Abd-B* promoter [134]. Do insulators therefore have a dual function in targeting regulatory domains to specific Hox promoters? Maybe not, because the insulator and long-range regulation functions of *Hox* boundaries are separable. Whereas the insulator activity of *Hox* boundaries relies on IBPs and can even be recapitulated by synthetic multimerized IBP binding sites [130,131], the long-range regulation activity relies on distinct, not yet fully characterized factors [135]. This is an important reminder that when large genomic segments are tested for insulator activity, they can span more than IBP binding sites and include coupled yet separable activities.

#### 4.2.2. Developmental Roles of Fly IBPs

The developmental roles of many *Drosophila* IBPs have not yet been fully explored. Several IBPs with described mutants are essential for viability [21,22,136,137,138,139,140,141]. Many questions remain regarding which and how many target genes IBPs regulate by insulation, and whether IBPs have dedicated insulator activities or additional functions inside or outside of the nucleus – such as *su(Hw)* and Cp190 that may act as a transcriptional repressor [142] or activator [143,144] respectively, and Cp190 may additionally play a structural role as a centrosomal protein [145]. To date, partial knock-down experiments of IBPs in cultured cells have led to limited effects on gene expression [12,23,43,136].

The strongest evidence to date that IBPs insulate developmental genes is the fact that several IBP mutants show homeotic phenotypes due to altered *Hox* gene expression patterns. *CTCF* mutants completely lacking zygotically-expressed and maternally inherited CTCF develop until the late pupal stage [146]. Their most conspicuous morphological defects are abdominal transformations that arise because *Abd-B* is expressed at more homogeneous levels in different parasegments compared to wildtype [146]. This is consistent with the notion that CTCF is important to maintain the independence of *Abd-B* regulatory domains (Figure 6). Similarly, *Cp190* zygotic mutants mis-express *abd-A* in a body segment in which *Ubx* is normally expressed [141]. Cp190 may therefore be required for insulation of a different set of *Hox* boundaries than CTCF, or their functions may be partially redundant. The stronger homeotic phenotypes observed in *Hox* boundary deletions [129,147] compared to *IBP* mutant phenotypes have also suggested that IBPs function redundantly at *Hox* boundaries. *Ibf2* [23] and *mod(mdg4)* [141] mutants also have homeotic phenotypes, and *BEAF-32* mutants have been reported to mis-express *Hox* genes that control anterior body segment identity [148].

## 5. Conclusions

Gene insulation remains enigmatic compared to other paradigms of transcriptional regulation yet work reviewed here highlights its importance for animal development. Two main challenges to study the biological relevance of insulation in mammals are the facts that global perturbation of CTCF results in cell lethality, and that effects of perturbing individual CTCF binding sites can be masked by the robust nature of regulatory and topological interactions in the genome. The small effects on gene expression observed in several experiments have moderated our view of how impactful CTCF may be on gene regulation. Yet, recent genetic studies have demonstrated that loss of CTCF binding in specific genomic contexts leads to transcriptional and developmental defects. Insulators prevent enhancer-promoter interactions between certain elements that are close (at endogenous locations or brought into proximity through chromosomal rearrangements). Moreover, TADs can help regulatory elements and promoters overcome otherwise prohibitory linear distances between them to enable regulation. 

The requirement for insulation is thus conserved in flies and mammals, notably at *Hox* genes, which have maintained CTCF binding sites for hundreds of millions of years to help organize animal body plans [18]. In flies, global perturbation studies of IBPs will be useful to explore how widespread their effects on gene regulation and on genome topology are. To date, a mutant fly showing widespread ectopic enhancer-promoter interactions has not yet been described and it remains unclear whether a master-regulator of the specificity of regulatory interactions exists.

The conspicuous topological role of CTCF in mammals has led to the view that it controls transcription through chromosomal loop formation. This is supported by the fact that TADs correspond to both structural and gene regulatory units and by the TAD perturbation experiments reviewed here. The “loop model” also reconciles many of the activities exerted by insulators discussed in Section 3 and the seemingly contradictory functions of insulators of blocking some regulatory interactions and fostering others, with little reported specificity for regulatory element or promoter type. Yet, we do not fully understand how genome topology impacts transcription. Single cell analyses of TAD boundaries revealed that only a fraction of TAD boundaries exist in a given cell at a given time as they are probabilistic and not absolute features [153]. How could dynamic TAD boundaries maintain independent domains of gene regulation? Moreover, assuming that insulators prevent regulatory element-promoter communication by maintaining them separated in 3D space, what physical distances would be relevant? Recent studies highlight that enhancers and promoters communicate dynamically across surprising distances (~300 nm in flies and mammals), likely reflecting that transcription occurs in hubs containing multiple RNA polymerase II complexes and other regulatory factors [154,155,156]. In brief, the relevant physical distances and kinetics that insulators would need to impose to block enhancer–promoter communication have yet to be explored. Comparing and contrasting studies in flies and mammals, in which insulation is conserved but the molecular mechanisms possibly less so, will be interesting to understand whether the universal mode-of-action of IBPs is by modulating genome topology, or whether yet unexplored mechanisms exist to control gene regulatory interactions.
